# Ichthyo-diversity assessment of the Old Brahmaputra river, Bangladesh: present stance and way forward

**DOI:** 10.1016/j.heliyon.2020.e05447

**Published:** 2020-11-07

**Authors:** Abul Bashar, Md. Fazle Rohani, Md. Rois Uddin, Md. Sazzad Hossain

**Affiliations:** a Department of Aquaculture, Bangladesh Agricultural University, Mymensingh, 2202, Bangladesh; b Department of Geography and Environmental Science, Ananda Mohan College, Mymensingh, 2200, Bangladesh

**Keywords:** Fish diversity, The Old Brahmaputra river, Shannon index, Simpson index, Agricultural science, Animal science, Aquaculture, Biodiversity, Fishery management

## Abstract

The study was carried out to obtain information on the present status and trend of finfish diversity of the Old Brahmaputra river, Bangladesh. Samples were collected directly from a professional fishing boat caught by different nets, traps and hooks from January 2019 to December 2019. Together with 4 exotic species, a total of 49 species under 6 families were recorded. Though a biodiversity index of 3.65854 and a dominance index of 0.030929 represent the richness of ichthyo-diversity within the river, Synbranchiformes and Tetraodontiformes were not reported throughout the study period. Linear regression analysis showed a positive correlation between water height of the river and monthly abundance of the species found. Catch composition of catfishes and snakeheads slumped while barbs showed triumph over previous findings. A majority of fish recorded were within the least concern category according to IUCN (2015) but portions also belonged to critically endangered, endangered, and vulnerable categories as well. Therefore, conservation measures must be infixed in the Old Brahmaputra river to hold the fish diversity in a sustainable state.

## 1 Introduction

Bangladesh, one of the top-ranked countries in capture fisheries (3^rd^) & inland aquaculture (5^th^) in the world (FAO, 2018), produces vast amount of fishes and shellfishes (4.277 million MT in FY, 2017–18) every year where capture fisheries contributed about 28.45% of the total country production in 2017–18 (DoF, 2018). By far, fish is the most commonly consumed animal source aliment across all population groups with an average consumption rate of 21.90 kg/person/year in Bangladesh (DoF, 2018). Fish is an important diet staple which provides micronutrients, vitamins, antioxidants, and other macro-elements (Bogard et al., 2015) and accounts for nearly 60% of animal protein intake in Bangladesh (Belton and Thilsted, 2014; Belton et al., 2014; Bogard et al., 2015).

The freshwater ecosystems of Bangladesh are much enriched (Shamsuzzaman et al., 2017; Newaz and Rahman, 2019), supporting at least 265 finfish and 24 prawn species (DoF 2018). The Brahmaputra river, one of the largest rivers of Asia, stands in prime position among the rivers of Bangladesh. Among the 1,300 floral and faunal species niched by this rich freshwater ecosystem, about 600 are endemic to the Brahmaputra river basin (Kabir et al., 2012). Branching off from the Brahmaputra main stream near Jamalpur district, with less water flow than its former self, the Old Brahmaputra is relegated to a minor river and flows south-east for approximately 200 km towards the Meghna river in Kishoreganj district (Wikipedia, 2019). Outstanding physical attributes characterized by favorable soil condition, sufficient water flow and depth throughout the year, meteorological environment, and the richness of biodiversity made the Old Brahmaputra river basin as one of the most fecund ecosystems from the perspective of fisheries and aquaculture in Bangladesh (Sania and Nesar, 2016). In many cases, fishing in this river is the pivotal and only means of livelihood available for traditional and amateur fishing communities (Mahmud, 2013).

Due to climate change and anthropogenic degradation of aquatic ecosystem, aquatic biodiversity, their catches, and their sustainability have undergone an unexpected switching (Hossain et al., 2012; Belton and Thilsted, 2014; Hossain, 2014; Shamsuzzaman et al., 2017; Akhi et al., 2020); the Old Brahmaputra is not an exception in this trend. Moreover, various factors including the physicochemical parameters of water (pollution, water depth, temperature, and salinity), meteorological parameters, and food availability affect the distribution and diversity of fish species (Cheng et al., 2016; Cheng et al., 2019; Guo et al., 2018). Among these, water depth of the habitat is one of the most important factors and so, our present study focused only to evaluate the effect of water depth on species diversity of the Old Brahmaputra river. In addition, there are so many points in river centric development which are critically incompatible with other sectors, notably with agricultural farming, environment, forest, and water management. Furthermore, Lack of proper management, policy-legislations, unplanned drainage as well as flood protection systems, and irrigation development are accelerating the abridgement of the existing riverine biological resources including finfish (Hossain, 2014; Rahman, 2008).

Safety measures to protect these diverse fish species from extinction are an urgent need because safeguarding of diverse fish species in nature not only brings about economic and therapeutic benefits, but also allows human to experience natural aestheticism (Moyle and Leidy, 1992). Therefore, it is wise to take all the precious actions as immediately as possible to protect the river biodiversity from being lost forever. In order to maintain a healthy sustainable catch and to protect all the species from being extinct, conservation of biodiversity should be the primary goal.

The main focuses of the various conservation measures include protection of biodiversity, reduction of excessive fishing pressure, restoration of favorable ecological conditions as well as facilitation of reproductive performance of the organisms (Hiddink et al., 2008; Sutherland et al., 2009). The extent and intensity of the conservation actions are influenced not only by the present abundance and richness of biodiversity but also ecological consideration of the habitat (Meyer et al., 2014). Therefore; it is paramount that from the very beginning, we have to understand the present status of fish diversity for the efficient application of the rational management actions in near future. Accordingly, the present study is undertaken to assess the diversity and abundance of finfish in the Old Brahmaputra river comparing our data with earlier studies.

## 2 Materials and methods

### 2.1 Ethical issue

The design and execution of the experiment were approved by the Ethical Committee of Bangladesh Agricultural University Research System (BAURES) upon meeting their guidelines.

### 2.2 Study area

**Figure 1:**
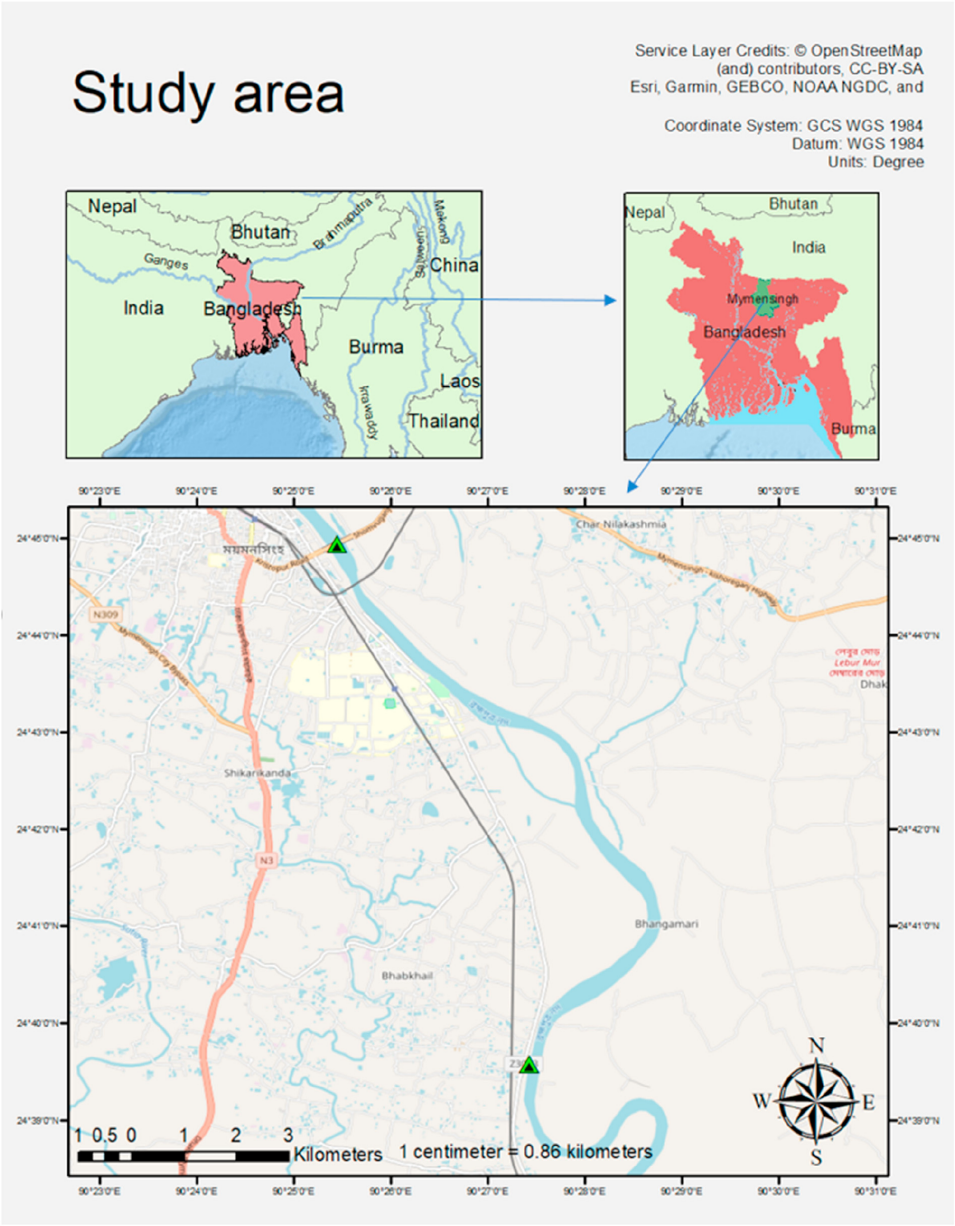
Study area specified by the distance between two triangular marks, indicating Bridge area (24°44′ 56.46″ N and 90° 25′ 27.5″ E) of Mymensingh city and Babukhali Bazar area (24°39′ 31.73″ N and 90° 27′ 28.1″ E).

This study was based on sample collection from the Old Brahmaputra river (Figure 1). Sampling area was defined from the Bridge area (24°44′ 56.46″ N and 90° 25′ 27.5″ E) of Mymensingh city to Babukhali Bazar area (24°39′ 31.73″ N and 90° 27′ 28.1″ E) for easy access of laboratory facilities of Bangladesh Agricultural University and also to facilitate further referencing as previous studies (Galib, 2015; Sania and Nesar, 2016; Raushon et al., 2017) were done in and adjacent to this part of the Old Brahmaputra.

### 2.3 Sample collection

Samples were collected fortnightly from a professional fishing boat at the time of fishing. A total of 1541 samples were collected by cast net (mesh size: 1 cm; 4 h hauling), seine net (mesh size: 0.5 cm; 2 h hauling), gill net (mesh size: 1 cm; deployed overnight), push net (mesh size: 0.5 cm; 4 h hauling), fishing traps (deployed overnight), and hooks and lines (deployed overnight) from January 2019 to December 2019. Wide range of fishing gears of small mesh size were used to ensure the retention of all fishes irrespective of species and size. Data on unavailable fishes were verified using a non-structured survey with local fisher folks (n = 30).

### 2.4 Identification of species and conservation status

Though most of the collected samples were identified immediately on the spot, all samples were preserved in 10% formalin solution and transported to the Fish Nutrition Laboratory, Bangladesh Agricultural University (BAU) for further study. All samples were taxonomically identified in the laboratory, based on morphometric and meristic characteristics cited by Quddus and Shafi (1983), Quddus et al. (1988), Rahman (1989), Talwar and Jhingran (1991), Rahman (2005), Nelson (2006), Roy et al. (2007), and Ahmed at el. (2009). Nomenclature and conservation status of each fish were assigned according to BDFISH (http://en.bdfish.org) and IUCN (2015) respectively.

### 2.5 Water depth

Due to varied water depth across the river, at least 10 measurements were done from the same vertical line of the river. In the same way, water depths were measured with a wooden meter scale during each sampling day from 10 random points within the sampling site and recorded data were averaged in MS Excel (2010).

### 2.6 Biodiversity index

Fish diversity was measured by Shannon diversity index (Shannon and Weaver, 1963) calculated according to Eq. (1):

(1) H=−∑Pi(lnPi)

where, Pi= ratio of individuals of *i*th species divided by all individuals of all species.

Dominance index of species diversity was calculated by Simpson index (Magurran, 2004) according to Eq. (2):

(2) $$D=\overset{s}{\underset{n=1}{\sum }}\left(\frac{ni(ni-1)}{N(N-1)}\right)$$

where,ni = number of *i*th individuals in the species and Ni= total number of individuals.

### 2.7 Data analysis

The empirical data recorded from this study were computed in MS Excel after necessary error checking and corrections. Primary analysis for producing graphs and tables was accomplished in MS Excel. To find out whether there was any correlation between the water depth and species diversity, linear regression analysis between the water depth and number of species recorded was done using Microsoft Excel (2010).

## 3 Results

### 3.1 Ichthyofauna of the Old Brahmaputra and conservation status

Finfish abundantly occupy the prolific niches of this ecosystem. The overall finfish found in current study are abstracted in table (Table 1) with their local conservation status according to IUCN (2015). A total of 45 native finfish species were found belonging to 6 orders where Cypriniformes occupied the prime position with 16 native species. Perciformes with 13 species dominated over Siluriformes, Osteoglossiformes, Clupeiformes and Beloniformes that contained 11, 2, 2 and 1 species respectively.

**Table 1:** List of native finfish species recorded from the Old Brahmaputra river with their local conservation status (IUCN, 2015).

Orders	Local Name	Scientific Name	Conservation Status
Beloniformes	Kakila	*Xenentodon cancila*	Least concern
Clupeiformes	Chapila	*Gudusia chapra*	Vulnerable
	kachki	*Corica soborna*	Least concern
Cypriniformes	Bou Machh	*Botia dario*	Endangered
	Gutum	*Lepidocephalichthys guntea*	Least concern
	Mola	*Amblypharyngodon mola*	Least concern
	Catla	*Catla catla*	Least concern
	Mrigal	*Cirrhinus mrigala*	Least concern
	Kalibaush	*Labeo calbasu*	Least concern
	Darkina	*Esomus danricus*	Least concern
	Bata	*Labeo bata*	Least concern
	Bhangan	*Labeo boga*	Critically endangered
	Rui	*Labeo rohita*	Least concern
	Dhela	*Osteobrama cotio*	Near threatened
	Kanpona	*Aplocheilus panchax*	Least concern
	Sarpunti	*Puntius sarana*	Near threatened
	Jati-punti	*Puntius sophore*	Least concern
	Tit-punti	*Puntius ticto*	Vulnerable
	Chela	*Chela cachius*	Vulnerable
Osteoglosiformes	Chital	*Notopterus chitala*	Endangered
	Foli	*Notopterus notopterus*	Vulnerable
Perciformes	Nama Chanda	*Chanda nama*	Least concern
	Ranga Chanda	*Parambassis ranga*	Least concern
	Koi	*Anabas testudineus*	Least concern
	Poa	*Otolithoides pama*	Least concern
	Taki	*Channa punctata*	Least concern
	Shol	*Channa striata*	Least concern
	Raga	*Channa orientalis*	Least concern
	Bele	*Glossogobius guiris*	Least concern
	Sal baim	*Mastacembelus armatus*	Endangered
	Chikra	*Mastacembelus pancalus*	Least concern
	Napit koi	*Badis badis*	Near threatened
	Lal Kholisha	*Trichogaster lalius*	Least concern
	Kholisha	*Trichogaster fasciata*	Least concern
Siluriformes	Buzuri Tengra	*Mystus bleekeri*	Least concern
	Tengra	*Mystus vittatus*	Least concern
	Rita	*Rita rita*	Endangered
	Ghaura	*Clupisoma garua*	Endangered
	Shing	*Heteropneustes fossilis*	Least concern
	Magur	*Clarias batrachus*	Least concern
	Kajuli	*Ailia coila*	Least concern
	Bacha	*Eutropiichthys vacha*	Least concern
	Batasi	*Neotropius atherinoides*	Least concern
	Pabda	*Ompok pabda*	Endangered
	Boal	*Wallago attu*	Endangered

Among 1541 samples, 59.19% of fish species were within the least concern category (Table 2) while only one species was found to be critically endangered according to IUCN (2015).

**Table 2:** Local conservation category of finfish species recorded from the Old Brahmaputra river.

Conversation categories	Number of species found	Percentage (%)
Least concern	29	59.19
Vulnerable	04	8.16
Near Threatened	04	8.16
Endangered	07	14.29
Critically endangered	01	2.04
Not Evaluated	04	8.16

### 3.2 Exotic species

The Old Brahmaputra contains various exotic species due to its favorable environmental parameters like temperature, modest rainfall, sufficient water depth and flow, and abundant primary production etc. However, most were rarely available to fisher's net. A total number of 3 exotic species belonging to Cypriniformes and only one exotic silurid were recorded throughout the study period (Table 3).

**Table 3:** List of exotic species recorded from the Old Brahmaputra river with their local conservation status (IUCN, 2015).

Order	Local Name	Scientific Name	Conservation Status
Cypriniformes	Carpio	*Cyprinus carpio*	Not evaluated
	Silver carp	*Hypophthalmichthys molitrix*	Not evaluated
	Bighead Carp	*Aristichthys nobilis*	Not evaluated
Siluriformes	Sucker mouth	*Hypostomus plecostomus*	Not evaluated

### 3.3 Catch composition and biodiversity index

**Figure 2:**
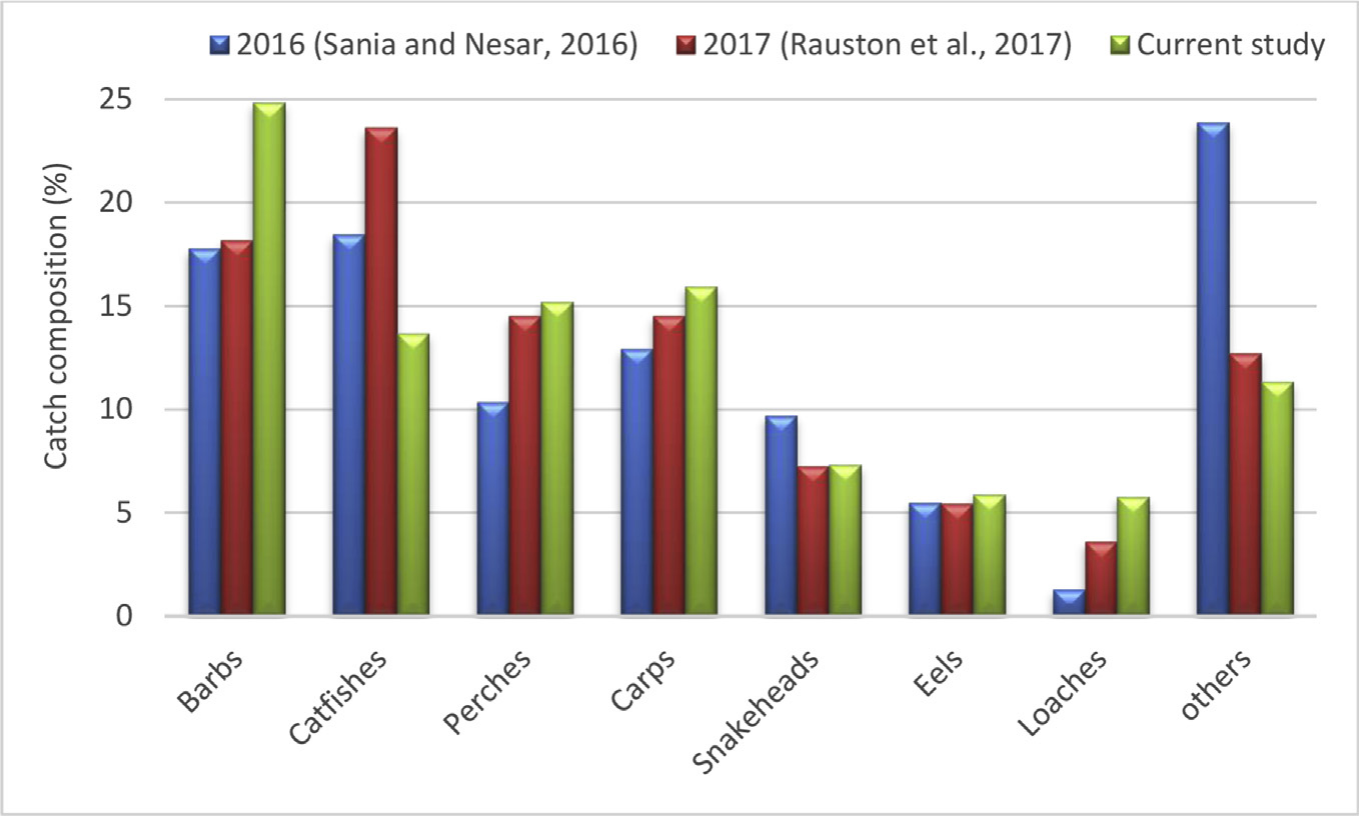
Comparison of catch composition of the Old Brahmaputra with previous findings.

*A. mola* was found to be greatest (6.34%) in community composition while *E. danricus* (4.67%) and *G. guiris* (4.09%) dominated over *O. pama* (3.37%) and *C. garua* (3.37%) (Table 4). Lowest abundance was recorded for *W. attu, R. rita,* and *H. plecostomus* with a catch composition of 0.195%. Our study revealed that catfishes and snakeheads decreased in the catch composition (%), however, barbs (mola, darkina, dhela, sarpunti, jatipunti, titpunti and chela), perches, carps, eels and loaches showed an increased catch rate when compared to the past findings (Sania and Nesar, 2016; Raushon et al., 2017) (Figure 2) which were obtained from survey based data collection using PRA tools from the same study area.

**Table 4:** Individual catch composition profile of finfish collected from the Old Brahmaputra.

Scientific Name	No. of fish (n*i*)	Portion of catch (P*i*)	% catch	ln (P*i*)	P*i* ln (P*i*)	$\frac{ni\left(ni-1\right)}{N\left(N-1\right)}$
*Xenentodon cancila*	37	0.0241038	2.401038	-3.72927	-0.08954	0.000561
*Gudusia chapra*	21	0.01362751	1.362751	-4.29566	-0.05854	0.000177
*Corica soborna*	26	0.01687216	1.687216	-4.08209	-0.06887	0.000274
*Botia dario*	42	0.02725503	2.725503	-3.60252	-0.09819	0.000726
*L. guntea*	47	0.03049968	3.049968	-3.49004	-0.10645	0.000911
*A. mola*	98	0.063595	6.359507	-2.75522	-0.17522	0.004006
*Catla catla*	26	0.016872	1.687216	-4.08209	-0.06887	0.000274
*Cirrhinus mrigala*	43	0.027904	2.790396	-3.57899	-0.09987	0.000761
*Labeo calbasu*	32	0.020766	2.076574	-3.87445	-0.08046	0.000418
*Esomus danricus*	72	0.046723	4.672291	-3.06352	-0.14314	0.002154
*Labeo bata*	23	0.014925	1.492537	-4.20469	-0.06276	0.000213
*Labeo boga*	8	0.005191	0.519143	-5.26075	-0.02731	2.36E-05
*Labeo rohita*	29	0.018819	1.881895	-3.97289	-0.07477	0.000342
*Osteobrama cotio*	33	0.021415	2.141467	-3.84368	-0.08231	0.000445
*A. panchax*	43	0.027904	2.790396	-3.57899	-0.09987	0.000761
*Puntius sarana*	8	0.005191	0.519143	-5.26075	-0.02731	2.36E-05
*Puntius sophore*	15	0.009734	0.973394	-4.63214	-0.04509	8.85E-05
*Puntius ticto*	110	0.071382	7.138222	-2.63971	-0.18843	0.005052
*Chela cachius*	37	0.02401	2.401038	-3.72927	-0.08954	0.000561
*Notopterus chitala*	5	0.003245	0.324465	-5.73075	-0.01859	8.43E-06
*N. notopterus*	7	0.004543	0.45425	-5.39428	-0.0245	1.77E-05
*Chanda nama*	62	0.040234	4.023361	-3.21305	-0.12927	0.001594
*Parambassis ranga*	35	0.022713	2.271252	-3.78484	-0.08596	0.000501
*Anabas testudineus*	26	0.016872	1.687216	-4.08209	-0.06887	0.000274
*Otolithoides pama*	52	0.033744	3.374432	-3.38894	-0.11436	0.001118
*Channa punctata*	58	0.037638	3.76379	-3.27974	-0.12344	0.001393
*Channa striata*	17	0.011032	1.10318	-4.50697	-0.04972	0.000115
*Channa orientalis*	38	0.024659	2.465931	-3.7026	-0.0913	0.000592
*Glossogobius guiris*	63	0.040883	4.088254	-3.19705	-0.1307	0.001646
*M. armatus*	33	0.021415	2.141467	-3.84368	-0.08231	0.000445
*M. pancalus*	58	0.037638	3.76379	-3.27974	-0.12344	0.001393
*Badis badis*	20	0.012979	1.297859	-4.34445	-0.05638	0.00016
*Trichogaster lalius*	23	0.014925	1.492537	-4.20469	-0.06276	0.000213
*T. fasciata*	17	0.011032	1.10318	-4.50697	-0.04972	0.000115
*Mystus bleekeri*	31	0.020117	2.011681	-3.9062	-0.07858	0.000392
*Mystus vittatus*	42	0.027255	2.725503	-3.60252	-0.09819	0.000726
*Rita rita*	3	0.001947	0.194679	-6.24157	-0.01215	2.53E-06
*Clupisoma garua*	52	0.033744	3.374432	-3.38894	-0.11436	0.001118
*H. fossilis*	18	0.011681	1.168073	-4.44982	-0.05198	0.000129
*Clarias batrachus*	11	0.007138	0.713822	-4.94229	-0.03528	4.64E-05
*Ailia coila*	14	0.009085	0.908501	-4.70113	-0.04271	7.67E-05
*E. vacha*	8	0.005191	0.519143	-5.26075	-0.02731	2.36E-05
*N. atherinoides*	9	0.00584	0.584036	-5.14296	-0.03004	3.03E-05
*Ompok pabda*	10	0.006489	0.648929	-5.0376	-0.03269	3.79E-05
*Wallago attu*	2	0.001298	0.129786	-6.64704	-0.00863	8.43E-07
*Cyprinus carpio*	25	0.016223	1.622323	-4.12131	-0.06686	0.000253
*H. molitrix*	32	0.020766	2.076574	-3.87445	-0.08046	0.000418
*Aristichthys nobilis*	28	0.01817	1.817002	-4.00798	-0.07283	0.000319
*H. plecostomus*	2	0.001298	0.129786	-6.64704	-0.00863	8.43E-07
Total (N)	1541				-3.65854	0.030929

In our current study, average Shannon index of biodiversity (H) was found to be 3.65854 while dominance index of Simpson (D) was calculated as 0.030929 (Table 4).

### 3.4 Correlation between water depth and diversity

In general, most of the fish were abundant in the Old Brahmaputra river for at least half of the year. Notwithstanding, *P. ticto* and *C. punctata* were found to be present throughout the year and *O. pabda*, *W. attu,* and *G. chapra* were abundant for only 3 months of the year.

**Figure 3:**
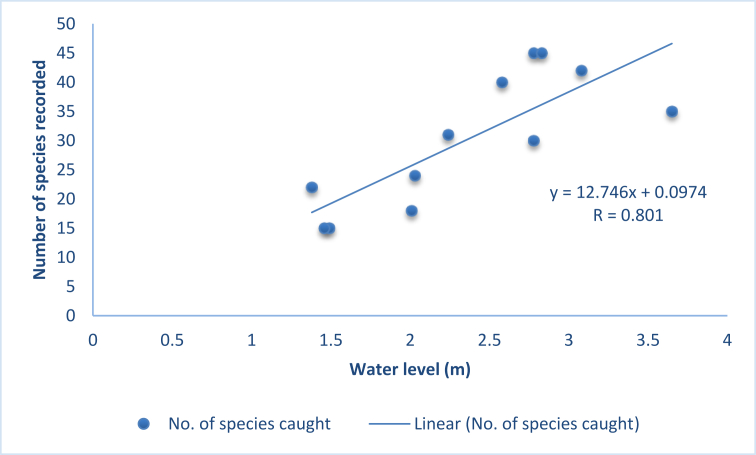
Correlation between number of species recorded and water depth of the Old Brahmaputra.

Table 5 shows the highest species diversity was found in April (42), while the lowest in October and November (15). Water level was found to be highest in July during heavy monsoon (3.65 m) and lowest during December (1.38 m). A linear regression graph between water depth and species diversity (Figure 3) shows that the value of correlation is 0.801 which interprets a strong positive relationship.

**Table 5:** Monthly average water depth and no. of species available.

Months	water level (m)	No. of fish species caught
Jan	2.03	24
Feb	2.24	30
Mar	2.58	38
Apr	2.78	42
May	2.83	41
Jun	3.08	34
Jul	3.65	32
Aug	2.78	28
Sep	2.01	17
Oct	1.49	15
Nov	1.46	15
Dec	1.38	21

### 3.5 Trend of biodiversity

**Figure 4:**
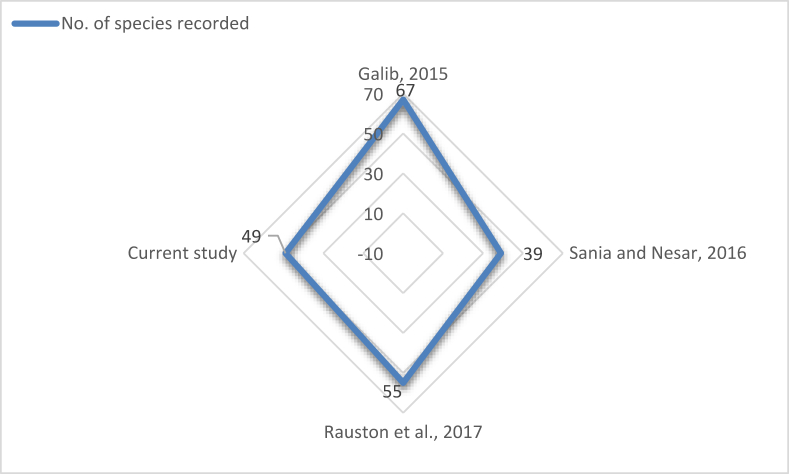
Fish species diversity trend of the Old Brahmaputra from 2009 to 2019.

Our study reported a total of 49 finfish species under 6 orders, whereas others reported 55 in 2017 (Rauston et el., 2017), 39 in 2016 (Sania and Nesar, 2016) and 67 in 2015 (Galib, 2015) (Figure 4).

**Figure 5:**
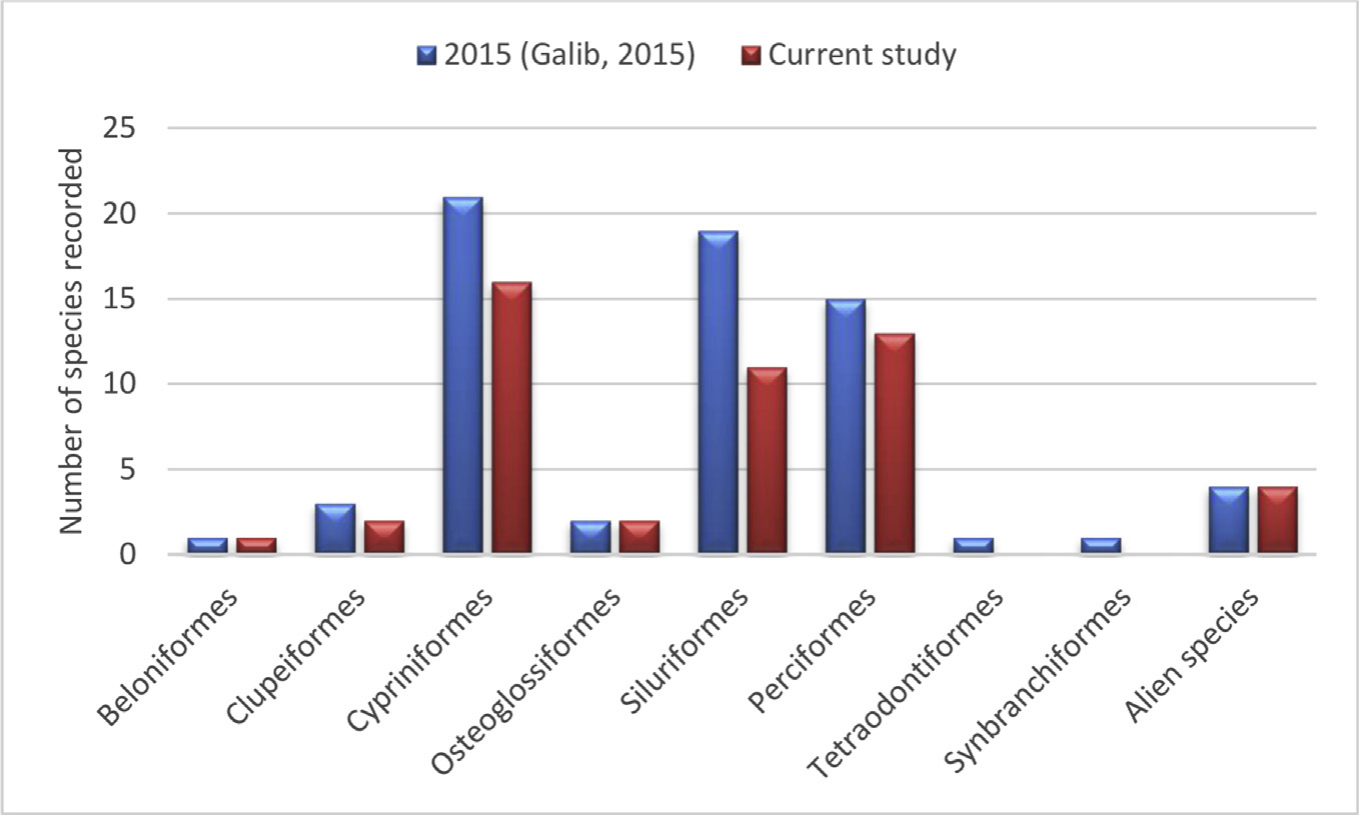
Comparison of number of species found under different orders from the Old Brahmaputra with previous study undertaken in 2015.

Figure 5 demonstrates that we were unable to detect any species under Tetraodontiformes and Synbranchiformes order from our study area while the number of species recorded decreased for Siluriformes, Perciformes, Cypriniformes and Clupeiformes. Unfortunately, no order in the Old Brahmaputra showed increasing trends in the number of species.

## 4 Discussion

The Old Brahmaputra is considered as an important haven for many aquatic species such as finfish, crustaceans, mollusks and other fauna and flora as well. Our study found 49 species of finfish belonging to 6 orders which is greater than the 39 species found by Sania and Nesar (2016) but less than the 55 species documented by Raushon et al. (2017) and the 67 species by Galib (2015). These findings illustrate the declination of finfish diversity in the Old Brahmaputra from 2015 that may be due to habitat alteration by anthropogenic activities, industrial pollution, siltation of the river course and adverse climatic condition as noted throughout the decade (Sania and Nesar, 2016). The breeding and nursery ground destruction along with heavy fishing pressure on stock made many of the species vulnerable to extinct (Galib, 2015).

Results show a belittled portion of critically endangered, minor portion of vulnerable and large portion of endangered and least concerned species. Most of the species under endangered categories niche the bottom ecological zone which interprets that we must endeavor the health of bottom ecosystem of the river. The only endangered species remains alive in the Old Brahmaputra river is *Labeo boga* which has switched so readily from its least concerned status (Dahanukar, 2010). Existence of this rapidly declined species in the Old Brahmaputra river is something optimistic and immediate conservation measures may ensure the restoration of this species from its ever worst running towards the extinction.

Catch composition data demonstrate a declination in catfish species, while populations of carps and barbs are in versus situation (Galib, 2015; Sania and Nesar, 2016; Raushon et al., 2017). Due to turn over of the benthic ecosystem by waste deposition from households, industries and cities of huge population (Ahmed et al., 2013; Bhuyan and Bakar, 2019) and due to lowered water depth, the catfishes, niching the bottom region affected mostly. In general, catfishes occupy higher position of food pyramid and prey on inferior species of food chain including littoral barbs and plankton eater smaller-sized carps (Gupta and Banerjee, 2014). As consequence of predatory catfish species abatement, food chain made the carps and barbs to increase in abundance (Shurin et al., 2002). However, delving more deeply from ecological consideration is required for conclusive remarks.

Catchability of fish depends on fishing efforts, hauling periods, ease of harvesting, water depths and water clarity etc (Mulazzani et al., 2015). In our current study, from January to May, the number of species available increased with the water depth. In June and July, due to heavy monsoon, water level increased and made the fishing more difficult. To avoid biasness in measuring diversity, fishing efforts were increased (150% for each gear) for these two months. In these months, small indigenous fishes and perches generally migrate to the nearby seasonal floodplains like beels and inundated rice fields from the main stream for reproduction purposes (Craig et al., 2014). This contributed to the lower species availability in this season. However, major carps (except common carps), catfishes, and loaches, demanding riverine environments for their reproduction (Rahman, 2008; Roy et al., 2018) were available in fishing nets. As water level decreased from August to November, small pelagic fish species, carps, and perches became less abundant while loaches, snakeheads, and eels increased in abundance. In December, though the water level was at its lowest, abundance of fish was higher in fisher's nets as lower water made fish easier to catch.

It is well established that the fish diversity and abundance is affected by a number of habitat variables, including water depth of marine (Piacenza et al., 2015), freshwater riverine (Degani et al., 1993; Mohapatra et al., 2007; Huang et al., 2019; Uttam et al., 2013; Guo et al., 2018) and coastal (Hossain et al., 2012) ecosystems. The average water depth recorded in this study throughout the study period is much less than the data recorded by Ahmed et al. (2013). However, in our present study linear regression analysis interprets that there is a strong positive correlation between water depth and the number of species available in Brahmaputra. This suggests stream excavation for desiltation as one of the conservation measures in the Old Brahmaputra river.

The trend of diversity represents a cluttered condition for the Old Brahmaputra river. In 2015, the number of species recorded was 67 (Galib, 2015), which dropped to only 39 in 2016 (Sania and Nesar, 2016). However, much improvement was noticed in 2017 with 55 species (Raushon et al., 2017). This might result from setting up a *Matsyarani* fish sanctuary (2009–2014) in the Old Brahmaputra river and execution of community-based management of riverine fisheries under the “Community-based Fisheries in Bangladesh: Bio-ecology, Production, Rights & Access, Governance & Replicability” project (FAO funded, 2018) by Faculty of Fisheries, BAU. These two actions can be traced as outstanding examples of management scheme that made a significant change towards restoration of the species which were in verge of extinction. In between these two management actions, in 2015, there was no management scheme for the Old Brahmaputra river and the stocks might be affected by overexploitation, explosive deadly fishing practices and fishing by dewatering according to fisher folk's perception. This verifies the decreased biodiversity observed in the following year by Sania and Nesar (2016). Within just a 2-years gap from 2017, the number of species has decreased to 49 according to current study. This suggests prolonged (not suspensive) management actions as a recommendation for the restoration of endangered and critically endangered species of the Old Brahmaputra river.

Shannon index of 3.65854 indicates that diversity of ichthyofauna in the Old Brahmaputra river is rich enough while Simpson index of dominance indicates low level of dominance which is favorable for an ecosystem in terms of evenness as the dominance index is inversely correlated with biodiversity index (Morris et al., 2014). However, loss of some species which were available in near past according to Galib (2015) and Raushon et al. (2017) is the fact of concern from diversity consideration.

From order consideration, we have already lost Tertraodontiformes and Synbranchiformes which were available in 2015 and species diversity faced an abridgement in Cypriniformes, Perciformes and Siluriformes order. This indicates a serious threat for Perciformes, Cypriniformes, and Siluriformes populations of the Old Brahmaputra river ecosystem.

## 5 Conclusion

Comparing our findings with past studies, a conclusive remark that the biodiversity of Old Brahmaputra is undergoing a critical stage where conservation is a must if we do not wish to issue more fish into the red list can be wrapped. To protect catfish and perch species especially from being threatened by anthropogenic activities, we must enact conservation measures based on scientific findings in conjugation with all stakeholders and policy makers. However, this study warrants further more investigations to understand the deep insights emphasizing on ecological, environmental and climate considerations.

## Declarations

### Author contribution statement

Abul Bashar: Conceived and designed the experiments; Analyzed and interpreted the data; Wrote the paper.

Md. Fazle Rohani: Conceived and designed the experiments; Performed the experiments; Wrote the paper.

Md. Rois Uddin: Performed the experiments; Analyzed and interpreted the data; Wrote the paper.

Md. Sazzad Hossain: Conceived and designed the experiments; Analyzed and interpreted the data; Contributed reagents, materials, analysis tools or data.

### Funding statement

This research did not receive any specific grant from funding agencies in the public, commercial, or not-for-profit sectors.

### Data availability statement

Data included in article.

### Declaration of interests statement

The authors declare no conflict of interest.

### Additional information

No additional information is available for this paper.
